# Predictive value of cerebrospinal fluid Alzheimer’s disease biomarkers and neuropsychological measures for cognitive and motor outcomes in Parkinson’s disease

**DOI:** 10.1093/braincomms/fcag166

**Published:** 2026-05-07

**Authors:** Alessandro Scalese, Guido Maria Giuffrè, Giulia Di Lazzaro, Angelo Tiziano Cimmino, Francesco Musso, Michela Orsini, Davide Quaranta, Maria Gabriella Vita, Carla Piano, Paolo Calabresi, Camillo Marra, Anna Rita Bentivoglio

**Affiliations:** Neurology Unit, Fondazione Policlinico Universitario Agostino Gemelli IRCCS, Rome 00168, Italy; Department of Neuroscience, Catholic University of the Sacred Heart, Rome 00168, Italy; Neurology Unit, Fondazione Policlinico Universitario Agostino Gemelli IRCCS, Rome 00168, Italy; Department of Neuroscience, Catholic University of the Sacred Heart, Rome 00168, Italy; Memory Clinic, Fondazione Policlinico Universitario Agostino Gemelli IRCCS, Rome 00168, Italy; Neurology Unit, Fondazione Policlinico Universitario Agostino Gemelli IRCCS, Rome 00168, Italy; Department of Neuroscience, Catholic University of the Sacred Heart, Rome 00168, Italy; Neurology Unit, Fondazione Policlinico Universitario Agostino Gemelli IRCCS, Rome 00168, Italy; Department of Neuroscience, Catholic University of the Sacred Heart, Rome 00168, Italy; Neurology Unit, Fondazione Policlinico Universitario Agostino Gemelli IRCCS, Rome 00168, Italy; Department of Neuroscience, Catholic University of the Sacred Heart, Rome 00168, Italy; Memory Clinic, Fondazione Policlinico Universitario Agostino Gemelli IRCCS, Rome 00168, Italy; Clinical Psychology, Fondazione Policlinico Universitario A. Gemelli IRCCS, Rome 00618, Italy; Neurology Unit, Fondazione Policlinico Universitario Agostino Gemelli IRCCS, Rome 00168, Italy; Department of Neuroscience, Catholic University of the Sacred Heart, Rome 00168, Italy; Department of Psychology, Catholic University of the Sacred Heart, Milan 20123, Italy; Neurology Unit, Fondazione Policlinico Universitario Agostino Gemelli IRCCS, Rome 00168, Italy; Neurology Unit, Fondazione Policlinico Universitario Agostino Gemelli IRCCS, Rome 00168, Italy; Department of Neuroscience, Catholic University of the Sacred Heart, Rome 00168, Italy; Neurology Unit, Fondazione Policlinico Universitario Agostino Gemelli IRCCS, Rome 00168, Italy; Department of Neuroscience, Catholic University of the Sacred Heart, Rome 00168, Italy; Memory Clinic, Fondazione Policlinico Universitario Agostino Gemelli IRCCS, Rome 00168, Italy; Department of Psychology, Catholic University of the Sacred Heart, Milan 20123, Italy; Neurology Unit, Fondazione Policlinico Universitario Agostino Gemelli IRCCS, Rome 00168, Italy; Department of Neuroscience, Catholic University of the Sacred Heart, Rome 00168, Italy

**Keywords:** Parkinson’s disease, CSF biomarkers, Alzheimer’s disease, mild cognitive impairment

## Abstract

Cognitive impairment is a major non-motor feature of Parkinson’s disease (PD). Alzheimer’s disease CSF biomarkers—amyloid-β (Aβ42, Aβ42/40), phosphorylated tau (p-tau) and total tau (t-tau)—may influence both cognitive and motor outcomes, but their role in relation to Parkinson’s disease phenotype or progression remains unclear. Moreover, few studies have investigated these associations using extensive neuropsychological batteries. The aim of the study was to examine the associations between CSF Alzheimer’s disease biomarkers, domain-specific neuropsychological performance and motor impairment in Parkinson’s disease and to evaluate their predictive value for longitudinal changes. Seventy-eight Parkinson’s disease patients underwent extensive neuropsychological examination, along with motor evaluation and Alzheimer’s disease CSF biomarkers quantification. Fifty-two patients completed follow-up motor assessment and 43 repeated cognitive evaluation after 18–24 months. Correlation and regression models adjusted for demographic and clinical covariates were applied. Thirty-nine patients were classified as mild cognitive impaired (PD-MCI), mostly with multidomain impairment. Amyloid-β biomarkers were associated with poorer attention, working memory, executive and language performance. Tau proteins were inversely associated with motor scores. PD-MCI patients tended to experience greater motor decline than cognitive unimpaired subjects. Over time, higher p-tau and t-tau predicted global cognitive decline (MMSE), while lower Aβ42/40 predicted worsening in verbal memory and reasoning. Higher t-tau and lower baseline MMSE independently predicted greater motor deterioration. CSF Alzheimer’s disease biomarkers showed domain-specific relationships with cognitive function and predicted both neuropsychological decline and motor progression. Combining detailed neuropsychological profiling with Alzheimer’s disease biomarkers assessment may improve identification of high-risk patients and support integrated biological definitions of Parkinson’s disease.

## Teaser

Scalese *et al*. report that, in Parkinson’s disease, CSF Alzheimer’s disease biomarkers are associated with cognition, motor function and disease worsening over time. Subjects with mild cognitive impairment may be prone to experience greater motor decline.

## Graphical Abstract

**Graphical Abstract:**
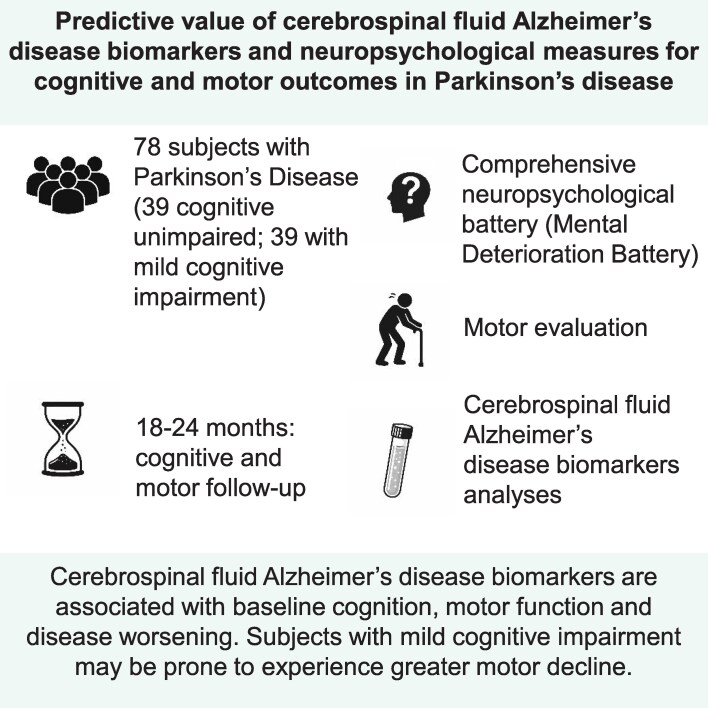

## Introduction

Cognitive impairment is one of the most important non-motor feature of Parkinson’s disease (PD), due to its impact on quality of life, disability, economic costs and caregiver burden.^1^ Patients with Parkinson’s disease have an almost 6-fold increased risk for developing dementia compared to healthy subjects.^2^ As seen in Alzheimer’s disease, the full spectrum of cognitive impairment occurs in Parkinson’s disease, ranging from subjective cognitive decline to mild cognitive impairment (PD-MCI) and dementia (PDD). Executive functions and memory are the most frequently affected cognitive domains.^3,4^ The prevalence of MCI at the time of diagnosis is around 20%, which increases to 40–50% after 5 years, more than the estimated prevalence of MCI in older population (60–90 years) of 16–20%. Despite some variabilities among studies, the prevalence of PDD is 17% at 5 years after diagnosis, 46% at 10 years and 83% at 20 years, whereas the global prevalence of dementia in the general population > 60 years ranges between 5 and 7%.^5^

Over the last decades, neurological diseases are increasingly being defined on the basis of biology through the use of biomarkers able to predict the disease pathology, phenotype and progression.^6-8^ The recent developments of ultrasensitive seed amplification assays have allowed the detection of α-synuclein (α-syn) *in vivo* with reliable accuracy,^9,10^ leading to the proposal of novel biological definitions of Parkinson’s disease, with CSF, blood, skin and extracellular vesicles showing the highest diagnostic performance in distinguishing Parkinson’s disease from control.^11,12^ The recent SynNeurGe and NSD-ISS research criteria have advised new definitions and staging systems for the disease, mainly based on biomarkers of synucleinopathy and striatal neurodegeneration.^13,14^ However, many individuals are more likely to have mixed pathology and comorbidities more than neuronal α-syn disease alone. Therefore, a key challenge for these novel definitions will be to take into account the potential impact of co-pathologies in shaping the clinical presentation and progression of disease. While CSF biomarkers for a biological diagnosis of Parkinson’s disease are still lacking a wide clinical application, CSF biomarkers of amyloidopathy (Aβ42 and Aβ42/40), tauopathy (phosphorylated tau—p-tau) and neurodegeneration (total tau—t-tau) have been widely used as measures of Alzheimer’s disease pathology *in vivo* and as predictors of cognitive impairment in Alzheimer’s disease.^15^

Several studies demonstrated that CSF biomarkers of Alzheimer’s disease pathology can predict cognitive impairment and motor progression in Parkinson’s disease as well. In particular, CSF Aβ42 levels showed consistently to correlate with outcomes of cognitive impairment^16,17^; meanwhile, there are controversial results showing reduced, normal or increased CSF levels of p-tau and t-tau associated to measures of cognition.^18^ Besides, CSF p-tau and t-tau have been found to correlate with worsening of motor symptoms, suggesting a potential role of tau pathology in accelerating motor progression.^19^ On the other hand, a recent retrospective study demonstrated that p-tau and t-tau could predict cognitive worsening in PD-MCI.^20^

The exact relationship between CSF biomarkers of AD and Parkinson’s disease phenotype or progression remains unclear. Moreover, only a few studies have assessed cognition in Parkinson’s disease patients using extensive neuropsychological evaluations. To contribute to this field, we analysed a cohort of patients with Parkinson’s disease who underwent a clinical evaluation, an extensive neuropsychological investigation and CSF analysis for Alzheimer’s disease biomarkers quantification. The present study aimed to prospectively assess the associations of cognitive status, motor impairment and symptoms progression with CSF biomarkers of Alzheimer’s disease in this cohort of Parkinson’s disease patients.

## Materials and methods

### Study population

Seventy-eight subjects with a clinically established diagnosis of Parkinson’s disease, according to the Movement Disorders Society (MDS) clinical diagnostic criteria,^21^ were enrolled from a consecutive series of native Italian-speaking patients evaluated at the outpatient clinic of the Fondazione Policlinico A. Gemelli IRCCS in Rome. Demographic and clinical data were collected. All subjects underwent a clinical evaluation in the best ON-medication state by a neurologist expert in movement disorders, an extensive neuropsychological investigation assessing different cognitive domains and a lumbar puncture to collect CSF samples. All human subjects provided informed consent. Our study was approved by the Ethical Committee of Fondazione Policlinico Universitario Agostino Gemelli IRCCS and was conducted in accordance with the 1964 Declaration of Helsinki and its later amendments.

Motor impairment and disease stage were assessed by means of MDS Unified Parkinson’s Disease Rating Scale (MDS-UPDRS parts I-IV)^22^ and Hoehn and Yahr staging scale.^23^ Concerning neuropsychological assessment, the Mini-Mental State Examination (MMSE)^24^ and an extensive version of the Mental Deterioration Battery were administered.^25^ After 18–24 months, subjects were evaluated during a follow-up visit. Motor impairment and disease stage at follow-up were assessed in 52 subjects. Follow-up neuropsychological assessments were available for 43 subjects.

MMSE was used to assess general cognition. The cognitive assessment comprised at least two neuropsychological tests for each of the five cognitive domains (attention/working memory, executive function, language, memory and visuospatial domains), allowing to enhance diagnostic sensitivity for PD-MCI according to the Level II diagnostic criteria for PD-MCI proposed by the MDS.^26^ To meet these criteria, at least two tests must be impaired, either within a single cognitive domain or across different cognitive domains. In the present population, a test was considered as impaired when the equivalent score was 0, indicating performance below the fifth percentile of the normative distribution after adjustment for age and education.

Attention/working memory domain was tested by digit span forward and spatial span forward.^27^ Executive functions were assessed by digit span backwards, spatial span backwards, Raven Progressive Matrices,^28^ Stroop colour word test^29^ and Trail Making Test (TMT).^30^ Language and lexical-semantic processing were studied using nouns denomination, phonological and semantic fluency tasks. Memory was evaluated in both visual and verbal modalities by the means of Rey-Osterrieth Complex Figure (ROCF) delayed reproduction^31^ and Rey Auditory Verbal Learning Test (RAVLT).^32^ Visuospatial skills were assessed through ROCF copy and Multiple Figure Target Cancellation (MFTC).^33^ Italian normative datasets were used to determine below the norm cut-offs.

### CSF analysis

CSF was collected, processed and stored according to literature-based recommendation and local standardized operating procedures.^34^ Lumbar punctures were performed in the morning after an overnight fast, using a 22-gauge Quincke spinal needle. The first 2 mL of CSF were collected in 15-mL polypropylene Falcon conical centrifuge tubes (catalogue number 62.554.001 Sarstedt, Nümbrecht, Germany) and used for routine analyses. Subsequently, 10-mL polypropylene tubes (catalogue number 62.610.201; Sarstedt, Nümbrecht, Germany) were used to directly collect 10 mL of CSF for Alzheimer’s disease biomarker quantification. These samples were immediately refrigerated and within 1 h of collection centrifuged at 2000×g for 10 min. CSF was then aliquoted in 0.5 mL polypropylene vials (catalogue number 72.730.006; Sarstedt, Nümbrecht, Germany) and stored at −80°C. CSF biomarkers of amyloidopathy (Aβ42 and Aβ42/40), tauopathy (p-tau) and neurodegeneration (t-tau) were measured within 30 days using a fully automated system based on a two-step sandwich chemiluminescent enzyme immunoassay on the Lumipulse instrument (Fujirebio), according to the manufacturer’s instructions (coefficients of variation: Aβ40: intra-assay ≤ 4.5%, inter-assay ≤ 4.5%; Aβ42: intra-assay ≤ 3.9%, inter-assay ≤ 4.6%; p-tau: intra-assay ≤ 5.0%, inter-assay ≤ 5.1%; t-tau: intra-assay ≤ 8.3%, inter-assay ≤ 7.6%). Analyses were performed in multiple batches following routine clinical procedures, using standardized calibration and quality control procedures. The cut-offs used were based on the manufacturer’s recommendations.^35^

### Statistical analysis

Statistical analyses were performed using ‘SPSS’ (‘Statistical Package for Social Science’, IBM SPSS Statistics. Armonk, NY: IBM Corp) v.28 for Mac. Subjects were classified according to the presence of cognitive impairment into two groups: subjects cognitively unimpaired (PD-CU) and subjects with mild cognitive impairment (PD-MCI). Subjects were also classified according to their AT(N) biomarker status.^6^ Descriptive statistics were used to illustrate clinical and demographic. Group differences between PD-CU and PD-MCI were assessed using the Mann–Whitney U test for continuous variables and the *χ*² test for categorical variables. Effect sizes were estimated using Cramer’s V for categorical variable (sex) and rank-biserial *r* for continuous variables, with 95% confidence intervals. Correlations between CSF biomarkers, neuropsychological tests and motor scores were investigated using Spearman correlation coefficient. Significant associations were further confirmed through linear regression analyses, adjusting for relevant covariates: for neuropsychological scores, models were adjusted for age, sex and education, while for motor scores, models were adjusted for age, sex, disease duration and L-Dopa Equivalent Daily Dose (LEDD). In order to assess longitudinal changes, the Wilcoxon signed-rank test was employed to compare baseline and follow-up data. Changes in cognitive and motor function over time were expressed as percentage changes in scale scores. Associations between CSF biomarkers and these longitudinal changes were analysed using Spearman correlation coefficient, followed by regression models adjusted for relevant covariates (age, sex and education for cognitive scores; age, sex, disease duration and change in LEDD for motor scores). For linear regression analyses, standardized regression coefficients (β) and adjusted *R*^2^ were reported to quantify the strength of associations and the proportion of variance explained. To assess potential attrition bias, baseline comparisons were performed for motor and cognitive follow-up. Specifically, demographic and motor clinical characteristics were compared between patients who completed motor follow-up and those who did not, while demographic variables and neuropsychological test performances were compared between patients who completed cognitive follow-up and those who did not. Given the large number of clinical and neuropsychological outcomes and the exploratory nature of the study, correction for multiple comparisons was not applied, and findings should be interpreted with caution.

## Results

### Clinical-demographic features

A total of 78 patients were included in the study. The clinical and demographic characteristics of the overall population, as well as the subgroups, are summarized in Table 1.

**Table 1:** Demographic and clinical characteristics of the overall population and diagnostic subgroups

	Overall population	PD-CU	PD-MCI	Effect size (95% CI)	*P*-values
	(*n* = 78)	(*n* = 39)	(*n* = 39)		
Female	16 (20.5%)	7 (17.9%)	9 (23.1%)	0.06 (0, 0.28)	0.582
Mean age at baseline	60.1 (8.9)	57.6 (8.9)	62.8 (8.2)	−0.32 (−0.53, −0.07)	**0**.**016**
Education	12.7 (3.6)	13.1 (3.1)	12.4 (4.1)	0.09 (−0.17, 0.33)	0.512
Disease duration	6.6 (4.3)	6.8 (4.4)	6.5 (4.3)	0.04 (0.21, 0.29)	0.763
LEDD	535 (517)	564 (488)	505 (549)	0.10 (−0.15, 0.35)	0.441
Hoehn and Yahr	2.0 (0.4)	1.9 (0.5)	2.1 (0.4)	−0.14 (0.38, 0.12)	0.191
UPDRS at baseline	17.8 (8.7)	18.2 (9.0)	17.5 (8.4)	0.01 (−0.24, 0.26)	0.948
Aβ42	886 (353)	924 (363)	847 (324)	0.11 (−0.14, 0.36)	0.390
Aβ42/40	0.093 (0.015)	0.094 (0.015)	0.093 (0.015)	0.05 (−0.21, 0.29)	0.735
P-tau	31.3 (13.1)	32.8 (14.6)	29.9 (11.3)	0.09 (−0.17, 0.33)	0.503
T-tau	247 (126)	253 (146)	242 (102)	−0.02 (−0.27, 0.23)	0.865

Continuous variables are presented as mean ± standard deviation (SD) and catego*r*ical variable (female) as *n* (%). Group differences between PD-CU and PD-MCI were assessed using t-tests or *χ*² tests, as appropriate. *P*-values refer the results of comparisons between the two groups. Effect sizes were reported utilizing Cramer’s V for categorical variable and rank-biserial r for continuous variables, along with 95% confidence intervals (CI). Bold font indicates statistical significance (*P* < 0.05). PD-CU: PD-cognitive unimpaired; PD-MCI: PD-mild cognitive impairment; LEDD: L-Dopa Equivalent Daily Dose; MDS-UPDRS-III: MDS Unified Parkinson’s Disease Rating Scale—Part III.

### Neuropsychological results

As seen in Table 1, 39 subjects were classified as PD-CU and 39 as PD-MCI. Four of them were PD-MCI single domain–amnestic type, while the other thirty-five subjects presented multidomain impairment, affecting different cognitive functions: attention/working memory (17/35, 49%), executive functions (20/35, 57%), language (26/35, 74%), memory (30/35, 86%) and visuospatial skills (20/35, 57%). As expected by the study design, cognitive performances were significantly lower across all domains in the PD-MCI group. Comprehensive neuropsychological results for the overall sample and each group are provided in Table 2.

**Table 2:** Neuropsychological results of overall population and subgroups at baseline

	Overall population	PD-CU	PD-MCI	Effect size (95% CI)	*P*-values
	(*n* = 78)	(*n* = 39)	(*n* = 39)		
MMSE	28.2 (2.0)	29.1 (1.0)	27.3 (2.4)	0.50 (0.29, 0.67)	**<0**.**001**
RAVLT imm.	35.0 (11.6)	41.2 (8.2)	28.8 (11.3)	0.66 (0.49, 0.79)	**<0**.**001**
RAVLT del.	6.7 (3.5)	8.4 (2.7)	4.9 (3.4)	0.57 (0.36, 0.72)	**<0**.**001**
RAVLT acc.	0.89 (0.10)	0.92 (0.06)	0.82 (0.13)	0.47 (0.25, 0.65)	**<0**.**001**
DS forward	5.6 (0.9)	5.8 (0.9)	4.4 (1.1)	0.17 (−0.08, 0.41)	0.162
DS backward	3.9 (1.2)	4.4 (1.1)	3.5 (1.2)	0.34 (0.09, 0.54)	**0**.**008**
SS forward	4.7 (1.1)	5.1 (0.9)	4.4 (1.1)	0.33 (0.09, 0.54)	**0**.**007**
SS backward	4.0 (1.3)	4.6 (1.0)	3.5 (1.4)	0.46 (0.24, 0.64)	**<0**.**001**
Raven	27.8 (6.2)	30.8 (3.7)	24.8 (6.8)	0.55 (0.35, 0.71)	**<0**.**001**
MFTC acc.	0.94 (0.06)	0.96 (0.04)	0.92 (0.07)	0.38 (0.14, 0.58)	**0**.**008**
MFTC time	58.1 (26.4)	46.7 (14.6)	69.7 (30.6)	−0.50 (−0.67, −0.26)	**<0**.**001**
TMT-A	61.7 (53.4)	36.5 (16.5)	82.2 (63.8)	−0.63 (−0.81, −0.35)	**0**.**001**
TMT-B	126.0 (84.1)	78.7 (28.6)	172.3 (96.1)	−0.67 (−0.84, −0.40)	**<0**.**001**
Phonological FT	34.5 (11.7)	39.2 (11.1)	29.8 (10.4)	0.45 (0.22, 0.63)	**<0**.**001**
Semantic FT	18.6 (6.0)	21.4 (5.4)	16.3 (5.6)	0.50 (0.28, 0.67)	**<0**.**001**
Denomination task	0.97 (0.05)	0.99 (0.02)	0.96 (0.06)	0.19 (−0.07, 0.42)	**0**.**023**
Stroop time	27.7 (19.8)	19.8 (6.7)	36.1 (25.0)	−0.48 (−0.66, −0.26)	**<0**.**001**
Stroop error	1.3 (3.6)	0.4 (0.8)	2.3 (5.1)	−0.24 (−0.47, 0.02)	**0**.**045**
ROCF copy	27.9 (9.6)	33.7 (2.0)	22.8 (10.6)	0.69 (0.50, 0.82)	**<0**.**001**
ROCF del.	12.7 (7.4)	17.5 (4.8)	8.5 (6.6)	0.73 (0.55, 0.84)	**<0**.**001**

*P*-values refer the results of comparisons between the two groups. Effect sizes were reported utilizing rank-biserial r, along with 95% confidence intervals (CI). Bold font indicates statistical significance (*P* < 0.05). PD-CU: PD-cognitive unimpaired; PD-MCI: PD-mild cognitive impairment. MMSE: Mini-Mental State Examination; RAVLT: Rey Auditory Verbal Learning Test (imm. = immediate recall, del. = delayed recall, acc. = accuracy); DS: digit span; SS: spatial span; MFTC: Multiple Figure Target Cancellation; TMT: Trail Making Test; FT: fluency task; ROCF: Rey-Osterrieth Complex Figure.

### CSF biomarkers

According to the AT(N) classification system, 53 patients were classified as A-T-N-; the others were classified as A+T-N- (14), A-T-N+ (5), A+T+N+ (3), A-T+N+ (1), A-T+N- (1), A+T-N+ (1).^6^ CSF biomarkers values are shown in Table 1. No statistically significant differences in CSF biomarkers values were found between the subgroups. A+ status was observed in 28.2% (11/39) of individuals in the PD-CU group and in 20.5% (8/39) of those in the PD-MCI group.

### CSF biomarkers in relation to neuropsychological assessment and MDS-UPDRS-III scores

**Figure 1:**
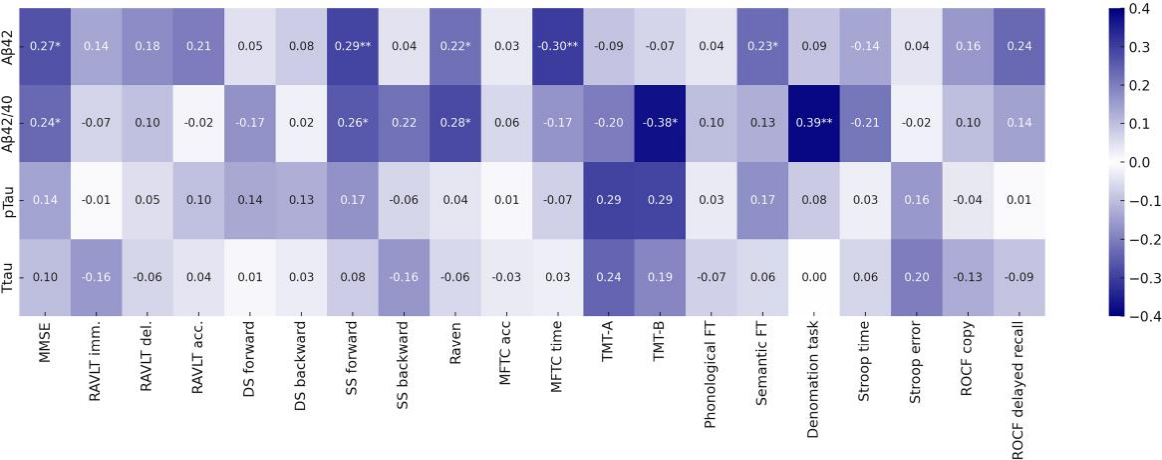
**Correlation heatmap between CSF biomarkers concentration and performances in neuropsychological assessments: the number in each cell represents the Spearman correlation coefficient (ρ).** The correlation analysis was carried out on the overall cohort (*N* = 78). At baseline, CSF amyloid measures and not CSF tau species predict cognitive performance. *P < 0.05; **P < 0.01. MMSE: Mini-Mental State Examination; RAVLT: Rey Auditory Verbal Learning Test (imm. = immediate recall, del. = delayed recall, acc. = accuracy); DS: digit span; SS: spatial span; MFTC: Multiple Figure Target Cancellation; TMT: Trail Making Test; FT: fluency task; ROCF: Rey-Osterrieth Complex Figure.

No statistically significant correlations were observed between CSF biomarker levels and either age or disease duration. Significant correlations between CSF biomarkers concentration and performances in neuropsychological assessments are illustrated in Fig. 1. These associations were further examined using linear regression analyses adjusted for age, sex and education. Lower CSF Aβ42 values were independently associated with poorer performance on MMSE (β = 0.244, t = 2.542, *P* = 0.013, adjusted *R*^2^ = 0.32), spatial span forward (β = 0.286, t = 2.648, *P* = 0.010, adjusted *R*^2^ = 0.14), Raven matrices (β = 0.211, t = 2.510, *P* = 0.014, adjusted *R*^2^ = 0.48) and MFTC time (β = −0.245, t = −2.274, *P* = 0.026, adjusted *R*^2^ = 0.14), while a lower CSF Aβ42/40 ratio was a significant independent predictor of a worse performance on spatial span forward (β = 0.241, t = 2.163, *P* = 0.034, adjusted *R*^2^ = 0.12), Raven matrices (β = 0.179, t = 2.066, *P* = 0.042, adjusted *R*^2^ = 0.47), TMT-B (β = −0.348, t = −2.140, *P* = 0.041, adjusted *R*^2^ = 0.28) and denomination task (β = 0.253, t = 2.187, *P* = 0.032, adjusted *R*^2^ = 0.06).

In contrast, CSF biomarkers of tauopathy and neurodegeneration did not show significant relationships with cognitive performance. However, both CSF tau proteins levels were inversely correlated with motor impairment at baseline, as measured by MDS-UPDRS-III (ρ = −0.235, *P* = 0.038; ρ = 0.255, *P* = 0.024, respectively). Aβ42 values, but not the Aβ42/40 ratio, also showed an inverse correlation with global motor impairment (ρ = −0.257, *P* = 0.023). These associations were reviewed in linear regression analyses adjusted for age, sex, disease duration and LEDD: p-tau (β = −0.283, t = −2.837, *P* = 0.006, adjusted *R*^2^ = 0.24), t-tau (β = −0.246, t = −2.424, *P* = 0.018, adjusted *R*^2^ = 0.22) and Aβ42 (β = −0.206, t = −1.972, *P* = 0.052, adjusted *R*^2^ = 0.20).

### CSF and clinical scores as biomarkers of disease progression

Baseline and follow-up data for subjects assessed at two time points are presented in Table 3. Overall, the cohort exhibited only minimal cognitive decline, with slight worsening observed in performance on Raven’s Matrices and phonological fluency task. In contrast, a more substantial decline in motor function was observed, reflected by a significant increase in MDS-UPDRS-III scores and higher LEDD requirements at follow-up. In the subgroup analysis (PD-CU and PD-MCI), patients with PD-MCI showed a trend towards greater motor deterioration compared to PD-CU, although with borderline statistical significance (*P* = 0.051 in PD-MCI versus *P* = 0.136 in PD-CU). Comparisons between participants who completed cognitive follow-up and those who did not showed no significant differences in demographic variables or baseline neuropsychological test performance. Similarly, comparisons of demographic and baseline clinical measures between participants who completed motor follow-up and those who did not revealed no significant differences, with the exception of baseline LEDD, which was higher in those who did not completed motor follow-up (U = 872, *P* = 0.038).

**Table 3:** Follow-up MDS-UPDRS-III and neuropsychological tests

	Baseline	Follow-up	*P*-value
		
MDS-UPDRS-III	16.6 (8.6)	18.9 (8.7)	**0**.**011**
LEDD	437 (419)	610 (463)	**<0**.**001**
MMSE	28.1 (2.0)	28.1 (2.0)	0.711
RAVLT imm.	34.2 (12.7)	35.9 (12.7)	0.258
RAVLT del.	6.7 (3.9)	6.3 (3.5)	0.423
RAVLT acc.	0.87 (0.11)	0.84 (0.17)	0.357
DS forward	5.7 (0.8)	5.7 (0.9)	0.545
DS backward	4.0 (1.1)	3.8 (1.0)	0.310
SS forward	4.6 (1.2)	4.5 (0.8)	0.465
SS backward	4.0 (1.3)	4.0 (0.9)	0.953
Raven	27.3 (6.2)	26.4 (6.9)	**0**.**004**
MFTC acc.	0.94 (0.05)	0.94 (0.06)	0.577
MFTC time	57.1 (23.9)	64.3 (32.1)	0.128
TMT-A	59.2 (35.7)	59.6 (61)	0.313
TMT-B	140.2 (92.7)	119.9 (70.1)	0.933
Phonological FT	34.5 (11.5)	32.3 (10.4)	**0**.**047**
Semantic FT	18.0 (5.3)	17.8 (5.2)	0.420
Denomination task	0.97 (0.04)	0.98 (0.04)	0.902
Stroop time	29.5 (20.2)	29.4 (18.5)	0.607
Stroop error	1.7 (4.7)	1.9 (4.5)	0.775
ROCF copy	27.2 (10.0)	29.2 (7.6)	0.467
ROCF del. recall	11.5 (7.5)	12.3 (6.8)	0.493

*P*-values refer to Wilcoxon signed-tank tests used to compare baseline and follow-up data. The MDS-UPDRS-III and LEDD measures were obtained from 52 subjects, while neuropsychological test results are available for 43 subjects. Bold font indicates statistical significance (*P* < 0.05). MDS-UPDRS-III: MDS Unified Parkinson’s Disease Rating Scale—Part III; LEDD: L-Dopa Equivalent Daily Dose; MMSE: Mini-Mental State Examination; RAVLT: Rey Auditory Verbal Learning Test (imm. = immediate recall, del. = delayed recall, acc. = accuracy); DS: digit span; SS: spatial span; MFTC: Multiple Figure Target Cancellation; TMT: Trail Making Test; FT: fluency task; ROCF: Rey-Osterrieth Complex Figure.

**Figure 2:**
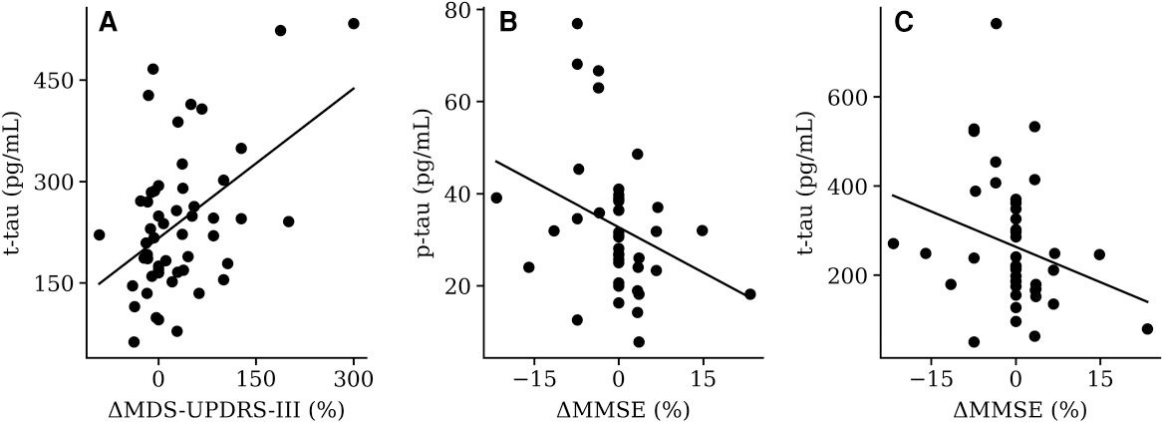
**CSF biomarkers and clinical variables changes at follow-up: scatter plots illustrating the most significant associations identified between CSF biomarkers and clinical variables changes at follow-up.** Each data point represents an individual patient included in the analysis. (**A**) Association between worsening of MDS-UPDRS-III scores (ΔMDS-UPDRS-III) and t-tau levels [*N* = 52; Spearman correlation, ρ = 0.32 (95% CI 0.05, 0.55), *P* = 0.020]; (**B**) worsening of MMSE scores (ΔMMSE) and p-tau [*N* = 43; Spearman correlation, ρ = −0.41 (95% CI −0.63, −0.12), *P* = 0.006]; (**C**) ΔMMSE and t-tau [*N* = 43; Spearman correlation, ρ = −0.37 (95% CI −0.60, −0.07), *P* = 0.016]. MDS-UPDRS-III: MDS Unified Parkinson’s Disease Rating Scale—Part III; MMSE: Mini-Mental State Examination.

Despite the modest cognitive changes observed at the group level, baseline concentrations of CSF Alzheimer’s disease biomarkers demonstrated significative predictive value for cognitive decline through time in specific neuropsychological measures. Specifically, higher baseline concentrations of CSF p-tau and t-tau were significantly correlated with greater deterioration in global cognition, as measured by change in MMSE (ρ = −0.410, *P* = 0.006; ρ = −0.366, *P* = 0.016, respectively) (Fig. 2). Additionally, a lower baseline Aβ42/40 ratio was significantly correlated with worsening in RAVLT immediate recall (ρ = 0.355, *P* = 0.020) and in Raven’s matrices performance (ρ = 0.318, *P* = 0.038). Furthermore, CSF p-tau levels were also significantly associated with decline in RAVLT accuracy (ρ = −0.336, *P* = 0.042).

Linear regression analyses, adjusted for age, education and sex demonstrated that Aβ42/40 ratio was independently associated with RAVLT immediate recall decline (β = 0.373, t = 2.411, *P* = 0.021, adjusted *R*^2^ = 0.10), while CSF p-tau was independently associated with MMSE worsening (β = −0.348, t = −2.260, *P* = 0.030, adjusted *R*^2^ = 0.05).

Concerning impairment in motor function, CSF t-tau baseline levels were found to be significantly associated with the percentage change in MDS-UPDRS-III (ρ = 0.322, *P* = 0.020) (Fig. 2).

In a first multiple linear regression model, including t-tau, age, sex, disease duration and change in LEDD as predictors, the model significantly predicted MDS-UPDRS-III worsening over time (F = 3.291, *P* = 0.013), explaining 19% of the variance (adjusted *R*^2^ = 0.19). Among the predictors, only t-tau emerged as a significant independent predictor of MDS-UPDRS-III worsening over time (β = 0.456, t = 3.670, *P* = 0.001).

In a second model, years of education and MMSE were added to account for potential effects of global cognitive status. This extended model remained significant (F = 3.224, *P* = 0.008) and explained 24% of the variance (adjusted *R*^2^ = 0.24), representing an improvement in the model fit compared with the first one. T-tau remained an independent predictor (β = 0.487, t = 3.604, *P* = 0.001). In addition, MMSE was significantly and inversely associated with the percentage change in MDS-UPDRS-III (β = −0.341, t = −2.156, *P* = 0.037).

## Discussion

We conducted an in-depth characterization of a cohort of Parkinson’s disease patients, integrating motor and cognitive assessments. All participants underwent a lumbar puncture, enabling the study of the associations between CSF biomarkers of Alzheimer’s disease and clinical features. Furthermore, a subset of our cohort was followed longitudinally, providing the opportunity to identify potential biomarkers of disease progression.

We characterized the motor and neuropsychological profile of our patients, divided in two groups, PD-CU and PD-MCI. The motor burden and CSF biomarkers values did not differ significantly between the two groups at the baseline, suggesting that MCI categorization is independent from those variables. Although language and memory deficits were the most frequently impaired domains, the PD-MCI subgroup lacked a clear cognitive signature. Consistent with our findings, previous studies have shown substantial interindividual variability, with heterogeneous patterns of impairment across executive, memory, language and visuospatial domains rather than a single predominant cognitive signature.^36,37^ A meta-analysis found that multiple domain subtype was the most common, present in two-thirds of PD-MCI cases.^38^ Another recent meta-analysis reported that tests assessing visual memory and visuospatial skills were particularly effective in distinguishing PD-CU from PD-MCI, while measures of executive functioning had the strongest ability to predict conversion from MCI to dementia.^39^ However, the neuropsychological characterization of our cohort extended beyond its purely classificatory and descriptive role. Patients with PD-MCI tended to experience greater motor decline compared with PD-CU, and, importantly, baseline global cognitive performance (measured by MMSE) emerged as an independent predictor of subsequent motor deterioration. These findings reinforce the need for more tailored and disease-specific assessment strategies to detect and characterize cognitive impairment across Parkinson’s disease continuum. Emerging tools—including targeted neuropsychological tests and machine learning approaches—may enhance the precision of diagnosis and improve the ability to monitor cognitive decline, potentially providing prognostic insights, even in relation to motor outcomes.^40,41^

In addition, relevant findings emerged concerning CSF Alzheimer’s disease biomarkers and clinical measures. Although the described associations account only for a limited proportion of the variance and do not imply causal links, they nonetheless provide meaningful insights into potential relationships between CSF Alzheimer’s disease biomarkers and clinical outcomes. Amyloidopathy biomarkers showed a significant association with impaired performance in attention, working memory, executive function and language domains. The association between amyloid pathology and cognitive impairment in synucleinopathies has been extensively investigated. Previous CSF studies have demonstrated a progressive decline in Aβ42 concentrations across diagnostic groups—healthy controls > PD-CU > PDD.^18,42^ However, β-amyloid PET imaging studies report similar rates of amyloid positivity in PD-MCI and PD-CU and a markedly higher prevalence in PDD, suggesting that, at least in the early stages of the disease, the cognitive decline in Parkinson’s disease is not driven by amyloidopathy alone.^43^ On the other hand, post-mortem studies showed that concomitant Alzheimer’s disease pathology in Parkinson’s disease patients is associated with increased amount of limbic and neocortical α-syn burden, suggesting that a causative relationship between the two pathologies might exist.^5,44,45^ Notably, the influence of amyloid pathology on Parkinson’s disease phenotypes is not only limited to cognition. Studies have linked elevated amyloid burden to the postural instability and gait difficulty subtype of Parkinson’s disease (PIGD).^46-48^ The mechanisms underlying gait control are more cholinergic-dependent and less associated with dopaminergic activity and amyloid may have a role in generating cholinergic deficits, damaging locomotor networks.^49^ In our cohort, CSF Aβ42 levels emerged as a predictor of global motor impairment at the baseline, with no association with PIGD phenotype.

While tau proteins were not associated with baseline cognitive performance, we observed negative correlations between CSF levels of p-tau and t-tau and motor function, as measured by MDS-UPDRS-III scores at baseline. Moreover, higher CSF values of t-tau were predictive of greater motor worsening over time, consistent with findings from previous studies.^50-53^ This observation supports the existence of some degrees of interaction between α-syn and tau proteins that follows a different neuropathological pathway compared to Alzheimer’s disease. Post-mortem studies have demonstrated the colocalization of tau and α-syn in Parkinson’s disease brains.^54,55^ Furthermore, genome-wide association studies have identified variants in the MAPT gene (that encodes the tau protein) as risk factors for developing Parkinson’s disease.^56^ Experimental models further support this interaction, suggesting that tau can enhance prion-like spread of α-syn, while tau knockout models attenuate α-syn propagation.^57^ It is known that α-syn and tau can interact in cells and their pathological conformations have the potential to template further misfolding and aggregation of each other. Moreover, α-syn and tau have a great number of shared interacting proteins, most of them involved in the cellular proteostasis. A defect in this shared interactome may be a pathological point of convergence, where aggregation of either α-syn or tau may indirectly trigger a loss of function of the other.^58^ Despite these mechanistic insights, the role of tau in Parkinson’s disease pathophysiology remains controversial and no concordant conclusion can be drawn. In our study, the association between higher CSF t-tau levels and greater motor worsening may reflect this putative ability of tau pathology to accelerate α-syn aggregation and striatal dopaminergic neurodegeneration. Alternatively, a primary tau-driven neurodegeneration, as seen in progressive supranuclear palsy and corticobasal degeneration, could offer a complementary hypothesis to our findings.^59^ It is important to highlight that, in our multiple linear regression model including both t-tau and MMSE, these variables independently predicted disease progression, suggesting that motor and cognitive deterioration may share partially overlapping pathological mechanisms.

Interestingly, we found that lower CSF tau proteins levels were associated with greater motor impairment at baseline. While other studies reported no association,^20,51,60^ observations from the PPMI cohort were consistent with our result.^47,61^ Moreover, several studies have shown that Parkinson’s disease patients exhibit lower levels of tau proteins than healthy controls.^50,61^ Therefore, as Zhang *et al*. suggested,^52^ one might expect that CSF tau levels would continue to decline rather than increase as the disease progresses. On the contrary, tau concentration (t-tau more than p-tau) tends to increase over time in Parkinson’s disease.^61^ This apparent contradiction underscores a fundamental difference in the interpretation of CSF tau biomarkers in Parkinson’s disease compared to Alzheimer’s disease. In Alzheimer’s disease, elevated CSF t-tau levels compared to controls are thought to reflect widespread neuronal damage and cell death, with tau being released from injured neurons. The axiom ‘more neurodegeneration, more CSF t-tau’, valid for Alzheimer’s disease, does not appear to hold true in Parkinson’s disease. One possible explanation is that, at least in the early stages of disease, tau may be trapped within neurons, either as a compensatory protective mechanism or due to its interaction with α-syn aggregates, thereby reducing its release into CSF.^62^ Furthermore, the seemingly paradoxical finding—higher baseline CSF t-tau being associated with less initial motor impairment, yet predicting greater motor worsening over time—could reflect disease heterogeneity. Elevated baseline tau might mark a biologically more aggressive subtype of Parkinson’s disease, where initial symptoms are mild but progression is rapid. Rather than serving as static indicators of disease severity, CSF tau levels may act as dynamic biomarkers reflecting underlying pathological activity and progression risk. This mechanistic hypothesis remains speculative and should be further investigated for future validation.

Finally, significant associations emerged between CSF biomarkers and longitudinal cognitive decline. Aβ42/40 was predictive of worsening of verbal memory span and general intelligence, while p-tau and t-tau were predictive of worsening in global cognition, as measured by MMSE. The role of CSF Alzheimer’s disease biomarkers in predicting cognitive deterioration in Parkinson’s disease has been examined in prior studies with mixed results. Most have reported associations between reduced CSF Aβ42 levels and cognitive decline, while tau protein levels were often not predictive.^50,63-66^ On the other hand, the DATATOP study found that elevated p-tau, rather than reduced Aβ42, was linked to cognitive worsening.^67^ Given that low Aβ42/40 and high p-tau are established core biomarkers of Alzheimer’s disease, these findings suggest that Alzheimer’s disease co-pathology may modulate cognitive trajectories in Parkinson’s disease. Notably, the robust association between p-tau and MMSE decline mirrors findings in Alzheimer’s disease, where tau pathology—not amyloid—is a stronger predictor of cognitive decline and conversion from MCI to dementia.^68,69^

The present study has potential limitations. First, the small sample size and the single-centre design might limit the generalizability of our findings, especially for the prospective part of the study. Secondly, the neuropsychological assessments used are not standardized for the diagnosis of MCI in Parkinson’s disease; however, at least two tests for each cognitive domain were used in order to have a high sensitivity in detecting PD-MCI. Third, the longitudinal analyses were based on a relatively short follow-up interval; a longer observation period would be helpful to better define disease trajectories and enable the identification of more sensitive prognostic markers. A further limitation is the incomplete longitudinal follow-up, which may have introduced attrition bias; however, baseline characteristics were largely comparable between participants who completed follow-up and those who did not. Finally, the absence of data about other biomarkers, such as α-syn or neurofilament light chain biomarkers, and the lack of imaging correlates limited the deeper characterization of associations between Alzheimer’s disease pathology, α-syn neuronal disease and other co-pathologies. Despite these limitations, a strength of the present study is the combination of detailed neuropsychological profiling with Alzheimer’s disease biomarker assessment, which may contribute to improved risk stratification for cognitive and motor decline and support the development of more integrated biological frameworks for Parkinson’s disease.

Overall, our findings highlight the clinical relevance of CSF biomarkers of Alzheimer’s disease in Parkinson’s disease, demonstrating their association with both cognitive and motor impairment, as well as their potential to predict disease progression. Complementarily, a comprehensive battery of neuropsychological tests allowed for the sensitive identification of PD-MCI patients, enabling improved classification and prognostic evaluation. These results support the notion that Alzheimer’s disease co-pathology contribute meaningfully to the clinical heterogeneity observed in Parkinson’s disease. As we move towards a biological definition of Parkinson’s disease, other significant factors besides synucleinopathy and striatal neurodegeneration should be taken into account. In line with recent updates to the AT(N) framework proposed by the Alzheimer’s Association, which now includes biomarkers of inflammation, vascular brain injury and α-synucleinopathy,^7^ a similar multidimensional approach may be warranted for Parkinson’s disease. Integrating CSF biomarkers of Alzheimer’s disease pathology in this evolving framework could enhance diagnostic precision, improve prognostic models and identify new therapeutic targets, serving as valuable additive elements to a more integrated and comprehensive biological definition of Parkinson’s disease.

## Data Availability

The datasets used during the current study are available from the corresponding author after a reasonable request.
